# The Association between Myocardial Iron Load and Ventricular Repolarization Parameters in Asymptomatic Beta-Thalassemia Patients

**DOI:** 10.1155/2012/170510

**Published:** 2012-05-15

**Authors:** Mehmet Kayrak, Kadir Acar, Enes Elvin Gul, Orhan Özbek, Turyan Abdulhalikov, Osman Sonmez, Hajrudin Alibaşiç

**Affiliations:** ^1^Department of Cardiology, Meram School of Medicine, Selcuk University, Meram, 42090 Konya, Turkey; ^2^Department of Hematology, Gazi School of Medicine, Gazi University, 06500 Ankara, Turkey; ^3^Department of Radiology, Meram School of Medicine, Selcuk University, 42080 Konya, Turkey; ^4^Department of Cardiology, Bezmialem Vakif University, 34093 Istanbul, Turkey

## Abstract

Previous studies have demonstrated impaired ventricular repolarization in patients with *β*-TM. However, the effect of iron overload with cardiac T2* magnetic resonance imaging (MRI) on cardiac repolarization remains unclear yet. We aimed to examine relationship between repolarization parameters and iron loading using cardiac T2* MRI in asymptomatic *β*-TM patients. Twenty-two *β*-TM patients and 22 age- and gender-matched healthy controls were enrolled to the study. From the 12-lead surface electrocardiography, regional and transmyocardial repolarization parameters were evaluated manually by two experienced cardiologists. All patients were also undergone MRI for cardiac T2* evaluation. Cardiac T2* score <20 msec was considered as iron overload status. Of the QT parameters, QT duration, corrected QT interval, and QT peak duration were significantly longer in the *β*-TM group compared to the healthy controls. *T*_*p*_ − *T*_*e*_ and *T*_*p*_ − *T*_*e*_ dispersions were also significantly prolonged in *β*-TM group compared to healthy controls. (*T*_*p*_ − *T*_*e*_)/QT was similar between groups. There was no correlation between repolarization parameters and cardiac T2* MRI values. In conclusion, although repolarization parameters were prolonged in asymptomatic *β*-TM patients compared with control, we could not find any relation between ECG findings and cardiac iron load.

## 1. Introduction

Beta-thalassemia major (*β*-TM) is a hereditary hemogolinopathy caused by impaired synthesis of *β*-globin chain and requires frequent blood transfusions [1]. In a consequence of transfusions, iron overload may develop. Deposition of iron in the heart may occur and cause severe cardiac complications such as heart failure and malignant arrhythmias [2]. The incidence of iron overload cardiomyopathy ranges from 11.4 to 15.1% in *β*-TM patients [3, 4]. Despite advances in chelating therapy, cardiovascular complications still remain main cause of mortality and morbidity in patients with *β*-TM [4]. 

Conventional QT parameters and transmyocardial repolarization parameters including *T*_peak_ − *T*_end_ interval (*T*_*p*_ − *T*_*e*_), dispersion ((*T*_*p*_ − *T*_*e*_)*d*), and (*T*_*p*_ − *T*_*e*_)/QT ratio have been used to predict cardiac arrhythmias in a number of clinical conditions such as Brugada syndrome and hypertrophic cardiomyopathy [5–7]. There are controversial results regarding conventional QT parameters in patients with *β*-TM. Although QT prolongation was demonstrated in patients with *β*-TM [8–10], there are studies with no statistical difference in the recent group [11, 12]. Chronic iron overload can lead to variety of arrhythmias including atrioventricular block, conduction defects, bradyarrhythmias, tachyarrhythmias, and sudden cardiac death (SCD) in patients with iron-overload cardiomyopathy [13–16]. Ulger et al. [9] investigated the role of ferritin on QT parameters in patients with *β*-TM and found no correlation of QT parameters with both serum ferritin levels and liver iron concentration. Interestingly, two experimental models have found that iron does not cause arrhythmias in guinea pig model and gerbil [17, 18]. Cardiac T2* MRI is more sensitive and specific method to predict iron loading than plasma ferritin levels and liver T2* measurement [19]. Cardiac T2* MRI identifies patients at high risk of heart failure and arrhythmia from myocardial siderosis in *β*-TM [20]. However, the relation of these repolarization parameters with cardiac T2* MRI value was not yet studied. The relationship between iron overload and electrocardiographic repolarization abnormalities remains controversial. Therefore, we aimed to investigate regional and transmyocardial repolarization parameters in *β*-TM patients and examine the relation of these repolarization parameters with cardiac T2* MRI value.

## 2. Material and Methods

### 2.1. Study Population

This is a cross-sectional case control study which was conducted at the Hematology department, Selcuk University, in Konya, Turkey. Twenty-two (14 men, mean age  26 ± 9  years) with diagnosis of *β*-TM and 22 age- and gender-matched healthy subjects were prospectively enrolled to the study. Included patients gave their verbal and written consent for participation in the study. The study was approved by the local ethical committee. The diagnosis of TM was made on hemogram, blood smear, hemoglobin electrophoresis, and clinical evaluation. The patients had been regularly transfused (every 3-4 weeks), and everyone received chronic chelation therapy (deferoxamine or deferiprone). Exclusion criteria were diabetes mellitus, history of coronary artery disease, congestive heart failure, congenital or acquired arrhythmia syndromes, use of drugs that lengthen the QT interval, current AF, bundle branch block, atrioventricular blocks, and unmeasurable T waves (<0.15 mV) on surface ECG. 

### 2.2. ECG Recording and Analyses

Surface 12-lead standard ECGs were recorded from each patient with a 50 mm/s paper speed at 10 mm/mV amplitude. Timing of electrocardiographic measurements was adjusted to the last blood transfusion, so ECG was performed at least two weeks later after blood transfusion. The ECGs were scanned at a 600 dpi resolution and analyzed manually by two experts who were blinded to the clinical status of the study population. Three consecutive ECG complexes were analyzed and given averaged measures for each lead.

 The QT intervals were measured from the onset of the first deflection of the QRS complex to the end of T wave in all 12 leads. If the T wave was flat or the end of the T wave was difficult to define, or if the T wave amplitude was <0.15 mV, the lead was excluded from measurement. The end of the QT interval was defined as the intersection of the terminal part of the T wave and the isoelectric line. If a U wave interrupted the T wave before it returned to baseline, the end of the QT interval was defined as the nadir between T and U waves.

Corrected QT interval (cQT) was calculated using Bazett's formula $\left(\text{cQT}=\text{QT}/\sqrt{R}-R\;\text{interval}\right)$. cQT dispersion (cQTd) was defined as the difference between maximum cQT and minimum cQT.

**Figure 1:**
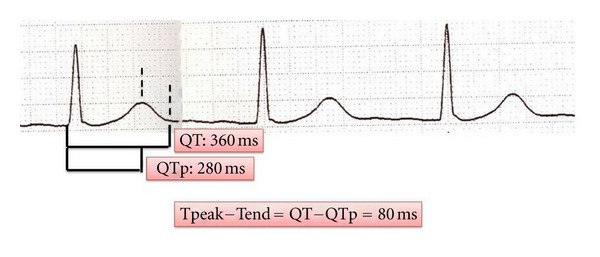
Measurement of *P* wave parameters on 12-lead surface ECG.

*T*_*p*_ − *T*_*e*_ interval was defined as the interval between QT and QT peak (*T*_*p*_ − *T*_*e*_ = QT − QTp). The difference between maximum *T*_*p*_ − *T*_*e*_ and minimum *T*_*p*_ − *T*_*e*_ was defined as *T*_*p*_ − *T*_*e*_ dispersion (Figure 1). The ratio of *T*_*p*_ − *T*_*e*_ to QT duration (*T*_*p*_ − *T*_*e*_/QT) was also calculated. Intra- and interobserver variablities for *T*_*p*_ − *T*_*e*_ were determined of 6.7% and 8.3%, respectively.

### 2.3. Cardiac Magnetic Imaging

Iron loading in the heart was evaluated with MRI T2*. MR images were acquired for all patients with a single imager equipped with a 1.5 T magnet (Siemens Symphony Erlangen, Germany). Body phased array coil to image a single 10 mm midventricular short axis slice at eight echoes times (ranging from 3 ms to 21 ms, increment 2.6 ms) with ECG gating and breath hold was also utilized. A gradient-echo sequence was used (flip angle 35, matrix 128 × 256). Double inversion recovery pulses were applied to suppress the blood signal and data was acquired every other cardiac cycle. The monoexponential decay model and the nonlinear curve fitting algorithm were used to fit the curve to obtain T2 measurement. We used CMR Tools software (http://www.cmrtools.com/) for quantification. 

### 2.4. Statistical Analysis

The statistical analysis was performed with the help of the Statistical Package for Social Sciences (SPSS for Windows) software (version 15.0) (SPSS Inc., Chicago, IL). The relation between the categorical variables was determined by the chi-square test. The distribution of the variables was analyzed with the Kolmogorov-Smirnow test. The Mann-Whitney *U* test was used for nonparametric comparison of two groups. A Spearman correlation test was used to assess linear association. The data were expressed as mean ± standard or median (inter quartile range) deviation according to the distribution properties, and *P* value under 0.05 was considered statistically significant.

## 3. Results

Table 1 summarizes the clinical, laboratory, and echocardiographic characteristics of the study population. *β*-TM patients were not different from the control group in age, gender, body-mass index, or blood pressure levels. The mean cardiac T2* value of patients with *β*-TM was 21.7 ± 9.0 ms. Hemoglobin (Hb) and hematocrit (Hct) levels were significantly lower in *β*-TM group than in the control group (Table 1). Heart rate was significantly increased in *β*-TM patients than controls (85 ± 8 bpm versus 78 ± 7 bpm, *P* = 0.004) (Table 1). Mean cardiac T2* MRI value was  23 ± 16 msec. 

**Figure 2:**
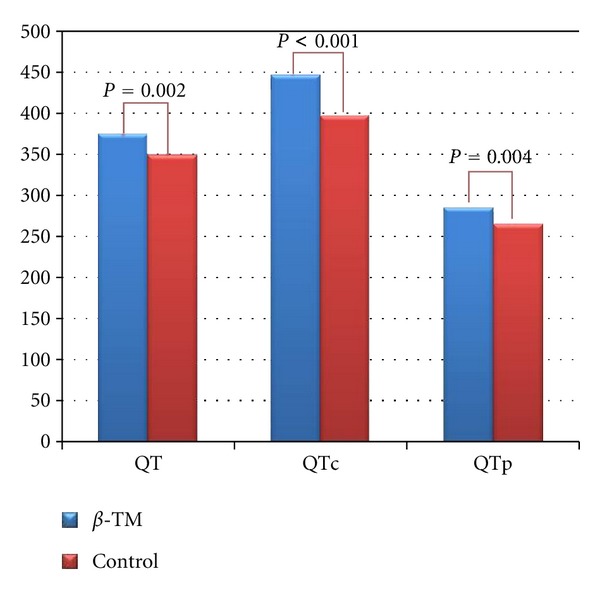
A comparison of QT, QTc, and QTp in patients with *β*-TM and healthy controls.

**Figure 3:**
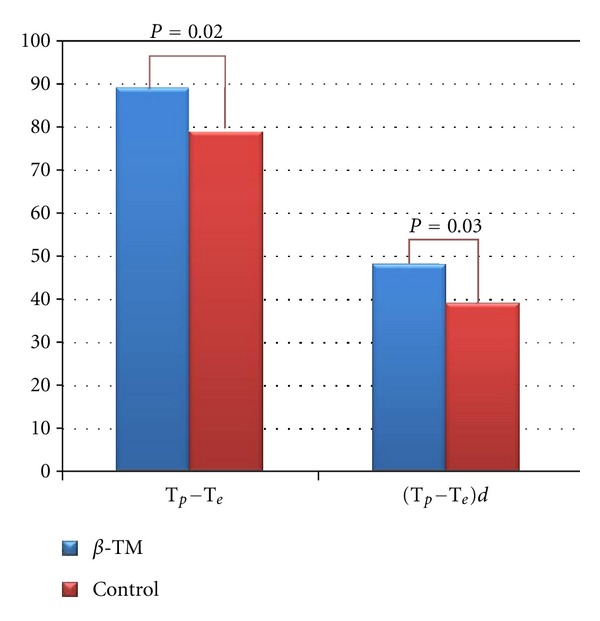
A comparison of *T*_*p*_ − *T*_*e*_ and (*T*_*p*_ − *T*_*e*_)*d* in patients with *β*-TM and healthy controls.

 Twelve-lead surface ECG analysis of repolarization parameters is listed in Table 2. With regard to regional QT parameters, compared with healthy controls, *β*-TM patients presented with increased QT duration (374 ± 25 ms versus 348 ± 26 ms, *P* = 0.002, resp.), QTc (445 ± 26 ms versus 396 ± 32 ms, *P* < 0.001, resp.), and peak QT duration (285 ± 22 ms versus 265 ± 27 ms, *P* = 0.009, resp.) (Figure 2). QTd and cQTd were comparable in two groups (*P* = 0.87 and *P* = 0.74, resp.). *T*_*p*_ − *T*_*e*_ and (*T*_*p*_ − *T*_*e*_)*d* were significantly higher in *β*-TM group than that in controls (*P* = 0.02 and *P* = 0.03, resp.) (Figure 3). No statistically significant difference was found in *T*_*p*_ − *T*_*e*_/QT ratio (*P* = 0.32) and T-wave amplitude (*P* = 0.39). Heart rate was significantly increased in patient group compared to control group (85 ± 8 bpm versus 78 ± 7 bpm, *P* = 0.004, resp.) (Table 2). 

ECG findings were similar in those patients with cardiac T2* < 20 msec and those with cardiac T2* ≥ 20 msec (Table 3). Both conventional QT and transmyocardial repolarization parameters did not show significant correlation with cardiac T2* MRI value (Table 4). 

## 4. Discussion

The present study demonstrated that repolarization parameters including QT, QTc, QTp, *T*_*p*_ − *T*_*e*_, and (*T*_*p*_ − *T*_*e*_)*d* were significantly prolonged in patients with *β*-TM compared to healthy subjects. Neither conventional QT parameters nor transmyocardial repolarization parameters were correlated with cardiac T2* MRI values. To the best of our knowledge, the present study is a first time study, where the relation between cardiac T2* MRI values and transmyocardial repolarization parameters was investigated.

 Cardiac complications, including arrhythmias, are among the leading causes of morbidity and mortality of transfusion-dependent, iron-overloaded *β*-TM patients [3]. Patients with chronic iron overload may develop a cardiomyopathy manifested by ventricular arrhythmias and heart failure [16]. Early detection of cardiac arrhythmias among transfusion-dependent *β*-TM patients is indicated for reduction of morbidity and mortality. The toxicity of iron was attributed to the following mechanisms: (a) excessive intracellular iron interferes with electrical function of the heart [21], (b) catalyze the generation of free radicals [22], and (c) cause selective dysfunction of Na^+^ channels [23]. Furthermore, iron overload may lead to apoptosis and fibrosis [2].

 Evaluation of conventional QT parameters was previously performed in *β*-TM patients. The results of these studies were conflicting. Some of them found prolonged QT, QTc, QTd, and cQTd in *β*-TM patients compared to controls [8–10]. In the contrary, there are two controversial studies which found conventional QT and dispersion parameters of no statistical difference between *β*-TM patients and controls [11, 12]. In our study, QT, QTc, and QTp were prolonged in patients with *β*-TM.

 The relationship between QT parameters and iron overload measured by serum ferritin level was investigated previously. Ulger et al. [9] found no correlation of QT parameters (QT, QTc, QTd, and cQTd) with both serum ferritin level and liver iron concentration in *β*-TM patients. There is only a study where Magri et al. [12] demonstrated that cardiac T2* MRI was significantly correlated with QT variability index but not with QTc. We also found that none of the conventional QT measurements were correlated with cardiac T2* MRI. We speculated that QT variability index may be more reliable to predict iron overload arrhythmias than conventional QT parameters. However, the measurement of QT variability index is very complex and requires more time records, additional software program, and complex formula. Therefore, clinic implication of QT variability parameters is limited.

Regarding transmyocardial repolarization, there are three layers in the myocardium: the endocardial, the M-cell, and the epicardial layer. The myocardial layers are at different repolarization phases creating transmyocardial nonhomogeneities, which become substrates for reentry causing malignant arrhythmias [24]. These transmyocardial nonhomogeneities are determined by the ECG parameters *T*_*p*_ − *T*_*e*_, (*T*_*p*_ − *T*_*e*_)*d*, and (*T*_*p*_ − *T*_*e*_)/QT ratio. Therefore we think that myocardial iron overload may contribute to creating transmyocardial nonhomogeneity and these ECG parameters may better demonstrate the relation between iron overload and repolarization parameters than conventional QT parameters. However, the data in *T*_*p*_ − *T*_*e*_ are very scarce in *β*-TM patients. There is only a study where Kocharian et al. [8] demonstrated prolonged *T*_*p*_ − *T*_*e*_ and (*T*_*p*_ − *T*_*e*_)*d* in patients with *β*-TM. Nevertheless, they did not investigate (*T*_*p*_ − *T*_*e*_)/QT ratio. We also found higher transmyocardial repolarization parameters except for (*T*_*p*_ − *T*_*e*_)/QT ratio in *β*-TM patients compared to the healthy subjects. In contrast to conventional QT parameters, there is no study investigating the relation between transmyocardial repolarization parameters and both ferritin levels and cardiac iron load. For the first time, we investigated the relation of transmyocardial repolarization parameters with cardiac T2* values. In our study, we found no difference in transmyocardial repolarization parameters in patients with T2* < 20  msec and those with T2* ≥ 20 msec. In addition, there was no linear relation between transmyocardial repolarization parameters and cardiac T2* values. These results support the theory that fibrosis as a cause of iron overload may be more deleterious than iron itself. Iron alone is not sufficient to cause arrhythmia and other comorbid conditions can contribute to arrhythmias. This issue was observed in nonhuman models [17, 18]. In addition, cardiac arrhythmias were not solely related to iron overload in patients with hemochromatosis [25]. Taken together, these data suggest that iron alone may be necessary but insufficient to cause cardiac arrhythmia in iron-overload conditions.

## 5. Limitations

A few limitations deserve mention: (a) this study is a cross-sectional study with small sample size and large prospective randomized studies are needed to clarify the relationship between the both impaired regional and transmyocardial repolarization parameters and subsequent cardiac events in *β*-TM patients, (b) we did not perform cardiac MRI in control group because of cost. In addition, because of controversial results in the literature, we aimed to evaluate whether repolarization parameters are impaired in patients with *β*-TM or not, (c) *β*-TM patients were under chelation therapy and this type of therapy may blind results of the study. Therefore our results may not be applicable to patients not undergoing this kind of therapy, (d) because of speculations that fibrosis as a cause of iron load may impair repolarization parameters, we think that investigation of noninvasive markers of fibrosis, such as metalloproteinase, procollagen type I, and procollagen type III needs to be investigated in future.

## 6. Conclusion

The present study demonstrated that repolarization parameters are impaired in asymptomatic *β*-TM patients and it was unrelated with cardiac T2* MRI values. Thus it needs to well-designed randomized investigations in this area.

## Figures and Tables

**Table 1:** Clinical, demographic, and laboratory characteristics of the study population.

	*β*-TM (*n* = 22)	Control (*n* = 22)	*P* value
Age (years)	26 ± 9	28 ± 7	0.47
Gender			
Female/male (*n*)	14/8	16/6	0.33
BMI (kg/m^2^)	23 ± 2.0	24 ± 1.6	0.44
SBP (mmHg)	122 ± 14	121 ± 16	0.36
DBP (mmHg)	81 ± 6	70 ± 7	0.22
Heart rate (bpm)	85 ± 8	76 ± 7	**0.004**
Hemoglobin (g/dL)	10.4 ± 1.3	13.3 ± 1.2	**0.001**
Hematocrit (%)	34.3 ± 6.6	44.2 ± 2.6	**0.001**
Cardiac T2* MRI (ms)	23 ± 16	—	—
Chelation therapy (%)	100	—	—

*β*-TM: *β*-thalassemia major; BMI: body-mass index; SBP: systolic blood pressure; DBP: diastolic blood pressure; MRI: magnetic resonance imaging.

**Table 2:** Electrocardiographic parameters of the *β*-TM group and controls.

	Controls (*n* = 22)	*β*-TM (*n* = 22)	*P* value
QT (ms)	348 ± 26	374 ± 25	**0.002**
QTd (ms)	40 ± 10	40 ± 16	0.87
QTp (ms)	265 ± 27	285 ± 22	**0.009**
(QTp)d (ms)	32 ± 15	31 ± 9.0	0.77
QTc (ms)	396 ± 32	445 ± 26	**<0.001**
cQTd (ms)	46 ± 12	48 ± 18	0.74
*T*_*p*_ − *T*_*e*_ (ms)	79 ± 12	89 ± 15	**0.02**
(*T*_*p*_ − *T*_*e*_)*d* (ms)	39 ± 11	48 ± 17	**0.03**
(*T*_*p*_ − *T*_*e*_)/QT	0.22 ± 0.03	0.23 ± 0.03	0.32
QRS duration (ms)	88 ± 14	93 ± 12	0.28
Heart rate (bpm)	78 ± 7	85 ± 8	**0.004**

*β*-TM: *β*-thalassemia major; QTd: QT dispersion; QTp: peak QT; (QTp)*d*: peak QT dispersion; QTc: corrected QT; cQTd: corrected QT dispersion; *T*_*p*_ − *T*_*e*_: difference between *T*-peak and *T*-end; (*T*_*p*_ − *T*_*e*_)*d*: *T*_*p*_ − *T*_*e*_ dispersion.

**Table 3:** Electrocardiographic findings of patients with *β*-TM according to cardiac T2* scores.

ECG parameters	T2* < 20 msec *n* = 12	T2* ≥ 20 msec *n* = 10	*P* value
QT (msec)	378 ± 28	387 ± 24	0.62
QTc (msec)	468 ± 35	478 ± 38	0.48
QTp (msec)	293 ± 25	291 ± 25	0.84
QTd (msec)	38 ± 11	40 ± 17	0.46
cQTd (msec)	48 ± 15	51 ± 17	0.59
*T*_*p*_ − *T*_*e*_ (msec)	85 ± 15	87 ± 13	0.57
(*T*_*p*_ − *T*_*e*_)*d* (msec)	47 ± 17	47 ± 16	0.94
*T*_*p*_ − *T*_*e*_/QT ratio	0.22 ± 0.03	0.24 ± 0.03	0.12
QRS duration (msec)	101 ± 19	99 ± 15	0.86
HR (bpm)	89 ± 11	92 ± 17	0.63

**Table 4:** Correlation and statistical significance of the ECG parameters to cardiac T2* values.

	*r*	*P*
QT	−0.17	0.46
QTc	−0.08	0.73
QTp	−0.29	0.18
QTd	−0.04	0.87
cQTd	−0.02	0.93
*T*_*p*_ − *T*_*e*_	−0.36	0.09
(*T*_*p*_ − *T*_*e*_)*d*	−0.04	0.86
*T*_*p*_ − *T*_*e*_/QT	0.31	0.14
